# Modes of Cell Death Induced by Photodynamic Therapy Using Zinc Phthalocyanine in Lung Cancer Cells Grown as a Monolayer and Three-Dimensional Multicellular Spheroids

**DOI:** 10.3390/molecules22050791

**Published:** 2017-05-16

**Authors:** Sello L. Manoto, Nicolette Houreld, Natasha Hodgkinson, Heidi Abrahamse

**Affiliations:** Laser Research Centre, Faculty of Health Sciences, University of Johannesburg, P.O. Box 17011, Doornfontein 2028, South Africa; lmanoto@csir.co.za (S.L.M.); nhoureld@uj.ac.za (N.H.); tashar@uj.ac.za (N.H.)

**Keywords:** photodynamic therapy, zinc phthalocyanine, monolayer, three-dimensional multicellular tumour spheroids

## Abstract

Photodynamic therapy (PDT) involves interaction of a photosensitizer, light, and molecular oxygen which produces singlet oxygen and subsequent tumour eradication. The development of second generation photosensitizers, such as phthalocyanines, has improved this technology. Customary monolayer cell culture techniques are, unfortunately, too simple to replicate treatment effects in vivo. Multicellular tumour spheroids may provide a better alternative since they mimic aspects of the human tumour environment. This study aimed to profile 84 genes involved in apoptosis following treatment with PDT on lung cancer cells (A549) grown in a monolayer versus three-dimensional multicellular tumour spheroids (250 and 500 μm). Gene expression profiling was performed 24 h post irradiation (680 nm; 5 J/cm^2^) with zinc sulfophthalocyanine (ZnPcS_mix_) to determine the genes involved in apoptotic cell death. In the monolayer cells, eight pro-apoptotic genes were upregulated, and two were downregulated. In the multicellular tumour spheroids (250 µm) there was upregulation of only 1 gene while there was downregulation of 56 genes. Apoptosis in the monolayer cultured cells was induced via both the intrinsic and extrinsic apoptotic pathways. However, in the multicellular tumour spheroids (250 and 500 µm) the apoptotic pathway that was followed was not conclusive.

## 1. Introduction

Lung cancer is the most commonly diagnosed cancer, representing 12% of all diagnosed cancers and 18% of cancer related deaths in both males and females, worldwide [1,2]. The most common predisposing factor to lung cancer development is cigarette smoking. In addition, exposure to radon, second hand smoke, and other diseases such as HIV are also factors that could lead to its development [3,4]. Being an aggressive cancer, the treatment of lung cancer has remained a challenging task. Conventional treatments include; surgery, radiation therapy, and chemotherapy. The type of therapy chosen is dependent upon the cancer type (small cell or non-small cell), development stage, and genetic characterization. Patients often undergo more than one type of treatment [5]. The development of targeted therapies is significantly changing the management of lung cancers. Such treatments include those that target presumed important molecules in cancer cell proliferation and survival [6].

Photodynamic therapy ‘PDT’ has emerged as an effective phototherapeutic modality used in the treatment of non-neoplastic and neoplastic diseases. PDT involves the use of light at a specific wavelength to selectively activate a photosensitizer ‘PS’ in the presence of molecular oxygen [7]. The activation of the PS results in the generation of singlet oxygen, or other reactive oxygen species (ROS), which causes oxidative damage to cellular components by damaging different biomolecules—including proteins, DNA, and lipids—and so leads to tumour cell death [8,9]. Although many PSs are being developed and used in targeted therapy, a ‘good’ PS should ideally be a single pure compound, it should have a strong absorption peak in the red to near-infrared spectral region (between 650 and 800 nm), it should possess a substantial triplet quantum yield, have no dark toxicity and a relatively rapid clearance rate from normal tissues [10].

Photofrin and hematoporphyrin are the most widely studied PSs in experimental and clinical trials [6]. Photofrin is clinically approved by the Food and Drug Administration ‘FDA’, of the United States of America, for the treatment of cancer. The main problems of this PS include long term photosensitivity, high degree of chemical heterogeneity, and poor absorption of tissue penetrating red light [11]. These downfalls have encouraged scientists to focus on research around the synthesis and testing of second generation PSs [11]. Among these are the phthalocyanines which have intense absorption in the visible red region, high efficacy in producing singlet oxygen, and ease of chemical modification and formation [12].

Traditionally, screening of drugs, for the treatment of cancer, has been conducted on conventional two dimensional ‘2D’ monolayer cultures of cells [13]. However, monolayer cultures, while useful for studying treatment effects, are too simple to replicate the many heterogeneous treatment effects found in vivo. Monolayer culture tests may provide misleading data which may not be easily translated to in vivo studies. In drug discovery research, approximately 10% of compounds progress successfully through clinical development, and many fail during clinical trials largely due to the lack of clinical efficacy and/or unacceptable toxicity. Additionally, a portion of these failures is attributed to data collected from the 2D monolayer culture tests where cellular responses to drug therapy are altered due to their ‘unnatural’ microenvironment [14].

Therefore, to bridge the gap between conventional monolayer cell studies and animal experiments, there is a need for the development of new cell culture models [15]. Multicellular tumour spheroids ‘MCTSs’ are three-dimensional ‘3D’ tumour cell aggregates and serve as an important model in cancer research for the evaluation of therapeutic interventions since they mimic different aspects of the human tumour environment [15,16]. These models provide valuable tools for in vitro identification of potential anticancer drug targets [16].

In non-cancerous cell cultures (normal cells), differences in phenotype and genotype when grown in 2D and 3D in vitro models have been documented. Researchers have identified that these differences are even more profound in cancer cells cultured in 3D when compared to a monolayer culture [17,18]. For experimental testing of the efficacy of new drugs in the treatment of cancer, it is essential to mimic in vivo environments as close as possible to yield accurate results, and the use of MCTSs can provide such environments.

The three key cell death pathways evoked by PDT are apoptosis, necrosis, and autophagy. Apoptosis is generally the main response to cells treated with PDT and in the ablation of tumours [11,19]. Stimuli responsible for triggering apoptosis include; tumour necrosis factor (TNF), DNA damage, ROS, and increased intracellular calcium levels. It is, however, unclear which of these pathways are triggered resulting in apoptotic cell death by PDT [20]. Studying the differential expression of genes involved in apoptosis could provide a clear understanding of PDT mediated cell death. In our previous study, it was demonstrated that photoactivated ZnPcS_mix_ induces apoptotic cell death, and that monolayer cells and MCTSs (250 and 500 µm) differ in their response to PDT, with MCTSs (250 µm) being the model most susceptible to PDT. The aim of the present study is to determine the genes involved in apoptosis mediated cell death following PDT, using the same parameters as our previous studies [16,21].

## 2. Results

### 2.1. Nuclear Morphology

**Figure 1:**
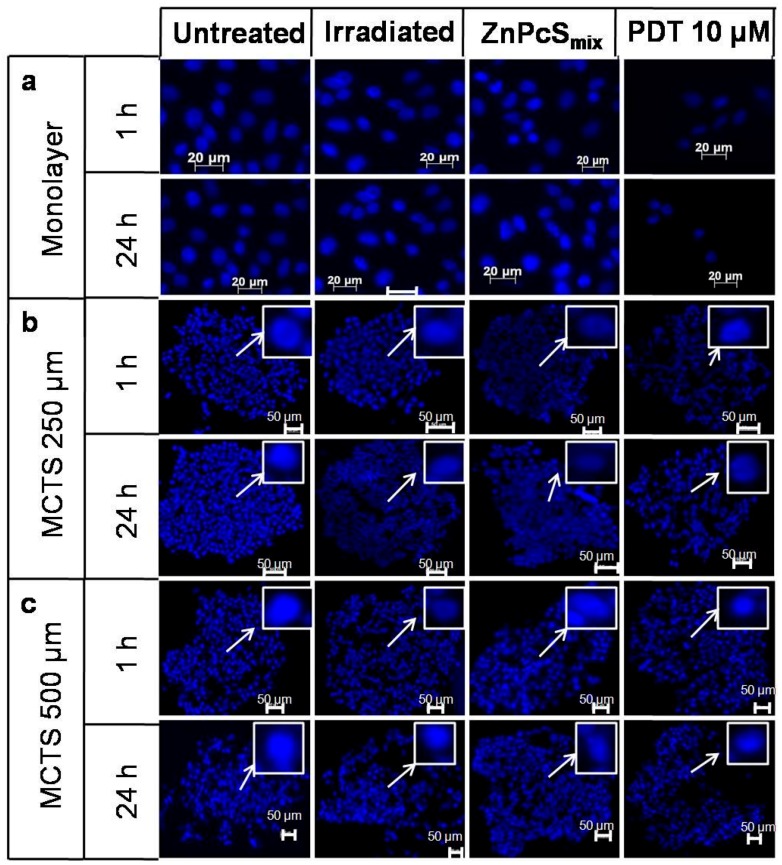
Fluorescence micrograph of monolayer cells (**a**), multicellular tumour spheroids (MCTSs) with a size of 250 μm (**b**) and MCTSs (500 μm) (**c**) stained with Hoechst 33342 1 or 24 h post incubation. Scale bar denotes 50 μm in MCTSs. The arrows show enlarged nuclei.

The Hoechst 33342 stain was used to evaluate apoptosis in the PDT treated cells (Figure 1). The monolayer cells treated with ZnPcS_mix_ (10 μM) or irradiated at 5 J/cm^2^ alone showed no changes in nuclei morphology and appeared similar to that of the untreated monolayer cells 1 and 24 h post incubation. Photoactivated ZnPcS_mix_ at 10 μM resulted in smaller numbers of nuclei, shrinkage of the nuclei, which is indicative of apoptosis, 1 h and 24 post PDT.

MCTSs (250 μm) irradiated with 5 J/cm^2^ or incubated with ZnPcS_mix_ (10 μM) alone showed no changes in nuclei morphology when compared to the untreated MCTSs 1 or 24 h post incubation. However, there was loss of cohesion in the MCTSs (250 μm) 1 and 24 h post PDT. No signs of cell damage, such as apoptosis and necrosis, were seen.

The nuclei of MCTSs (500 μm) irradiated with 5 J/cm^2^ or incubated with 10 μM of ZnPcS_mix_ alone appeared similar to that of untreated MCTSs, 1 or 24 h post PDT. These findings were similar to that of monolayer and MCTSs with a size of 250 μm. There was no loss of cohesion in MCTSs with a size of 500 μm 1 or 24 h post PDT. Similarly to that of MCTSs at 250 μm, there were no signs of apoptosis and necrosis.

### 2.2. Real-Time Reverse Transcription Polymerase Chain Reaction (RT-PCR)

Real-time RT-qPCR was used to determine the genes involved in apoptosis mediated cell death 24 h after PDT. The Human Apoptosis RT^2^ Profiler PCR Array profiles the expression of 84 key genes involved in programmed cell death. Photoactivated ZnPcS_mix_ (10 µM) in A549 monolayer cells resulted in a significant upregulation of the following 10 genes; ABL1, BAG3, BAK1, BCL2L10, BID, CASP5, GADD45A, HRK, TNF, and TP53BP2, and downregulation of three genes—namely: BIRC3, CASP3, and CASP6 (Table 1). Photoactivated ZnPcS_mix_ (10 µM) in 250 µm MCTSs showed upregulation of only 1 gene (BNIP3), while there was downregulation of 56 genes as described in Table 2. Six genes (BCL2L10, BNIP3, BNIP3L, CD40LG, and IL10) were upregulated, and three genes downregulated (BAX, TNFRSF11B, and TNFRSF21), when 500 µm MCTSs were treated with 10 µM ZnPcS_mix_ and irradiated with a wavelength of 680 nm at a fluence of 5 J/cm^2^ (Table 3).

## 3. Discussion

### 3.1. Gene Expression Profiling in the Monolayer Cultures

Two-dimensional monolayer cell cultures do not adequately represent an in vivo environment, while three-dimensional culture systems, such as MCTSs, better resemble in vivo tumour phenotypes, and are gradually becoming a more suitable model for the screening of anti-cancer drugs and development of new therapeutic interventions [22]. In our previous studies, we demonstrated that lung cancer MCTSs, with a size of 250 μm, were more susceptible to PDT than monolayer cell cultures [21], while MCTSs with a size of 500 μm were resistant to PDT [16]. The principal mechanism of PDT induced cell death in both studies was apoptosis. During PDT induced apoptotic cell death, expression of genes and their proteins are altered [20]. The present study used the SABiosciences Human Apoptosis RT^2^ Profiler PCR Array to determine the expression of 84 key genes involved in apoptosis in A549 cells grown as a monolayer and as MCTSs (250 and 500 μm) 24 h post PDT to determine the mechanisms involved in apoptotic cell death.

Gene expression profiling 24 h post PDT revealed differential genetic expression profiles in monolayer cells versus MCTSs (250 and 500 µm). These results suggest different mechanisms in the apoptotic cell death of A549 cells grown as a monolayer and as MCTSs. Gene expression profiling in monolayer cells revealed the upregulation of eight pro-apoptotic genes—namely ABL1, BAK1, BID, CASP5, GADD45A, HRK, TNF, TP53BP2—and downregulation of two pro-apoptotic genes (CASP3 and CASP6). Anti-apoptotic genes that were upregulated were BAG3 and BCL2L10, while BIRC3 was downregulated. From these results, it was clear that the genes which were predominantly over expressed were pro-apoptotic, correlating with the results found in our previous study, using Annexin V-FITC, which revealed apoptotic cell death in monolayer A549 cells post PDT [21].

Tissue necrosis factor ‘TNF’, which triggers the extrinsic apoptotic pathway, was upregulated in the monolayer cultured cells. TNF induces apoptosis by binding to its cognate receptors belonging to the TNF receptor ‘TNFR’ family [23]. This leads to the formation of cytosolic signalling complexes which initiate apoptosis signalling. Subsequently, Fas-associated death domain ‘FADD’ and inactive caspase-8 join, resulting in the formation of complex II. The presence of several molecules of caspase-8 in complex II leads to proximity activation of these signalling proteins. Activated caspase-8 cleaves and activates executioner caspases which in turn cleave multiple substrates resulting in apoptotic cell death [23,24]. Although TNF is involved in autophagy, deficient mechanisms can result in over activation c-Jun N-terminal kinase ‘JNK’, which increases caspase-8 activation, and allows for the initiation of TNF-dependent apoptosis.

BH3 interacting domain death agonist ‘BID’ is a pro-apoptotic gene that encodes a death agonist that heterodimerizes with either agonist BAX or antagonist BAK, BCL-2 family members. Activated BAX/BAK causes outer mitochondrial membrane permeabilisation ‘MOMP’, which results in the cytosolic release of apoptogenic factors—such as cytochrome c and Smac/DIABLO—that cause activation of the caspase cascade. A study conducted using zinc phthalocyanine PDT in human MCF-7c3 breast cancer cells demonstrated PDT induced BID activation [24].

BAG3 is a member of the BAG family of co-chaperones that influences cell survival by interacting with different molecular partners, and hence activating multiple pathways. BAG3 can potently suppress cell apoptosis via its interaction with Hsp70, a chaperone protein able to temper apoptosis by interfering with cytochrome c release, apoptosome formation, and other events involved in cell death. Recent reports have shown additional functions of this chaperone in the regulation of autophagy [25]. However, in this study, the upregulation of this gene was not sufficient in blocking apoptosis. BCL-2 antagonist/killer 1 ‘BAK1’, in the presence of an appropriate stimulus, may promote apoptosis by antagonizing the anti-apoptotic action of BCL-2 [26]. In our study, BAK1 upregulation resulted in the inhibition of BCL-2, thereby with subsequent apoptosis.

The growth arrest and DNA damage inducible gene 45 alpha GADD45α is a stress signal response gene that, although its exact mechanism is not fully understood, is involved in regulating DNA repair and apoptosis. The GADD45 family members interact with the upstream kinase MTK1/MEKK4 when exposed to environmental stresses, which results in apoptosis through the p38/JNK pathways [27]. A study by Yin et al. (2004) demonstrated apoptotic cell death in human MCF-7 breast cancer cells exposed to troglitazone when the GADD45 gene was upregulated [28]. Another study by Chang et al. (2013) demonstrated that 2-methoxyestradiol (2-ME), an oestrogen metabolite, induces mitochondrial mediated apoptosis by the upregulation of HRK. HRK is involved in the 2-ME–induced apoptotic pathway by activating caspase through BAK-mediated cytochrome c release [29]. Our present study yielded similar results in the A549 cells showing upregulation of GADD45 and subsequent apoptosis of cells.

Tumour protein p53 binding protein 2 ‘TP53BP2’ p53 induces the expression of genes such as BAX and other BCL-2 family members that promote the release of cytochrome c into the cytoplasm from mitochondria and initiate the intrinsic apoptotic pathway. Tumour protein p53, depending on its origin, however, may have various effects on the regulation of autophagy. Cytoplasmic p53 inhibits autophagy, while nuclear p53 activates autophagy. The present study demonstrated upregulation of TP53BP2 which codes for p53 (TP53BP2 specifically regulates p53-dependent apoptosis), in combination with other pro-apoptotic genes, and therefore inducing apoptosis [30]. Ye et al. (2015) demonstrated the role of p53 in apoptosis of MCF-7 cells [31], and the findings confer with what was demonstrated in our study, where p53 was upregulated and cells underwent apoptotic cell death.

**Figure 2:**
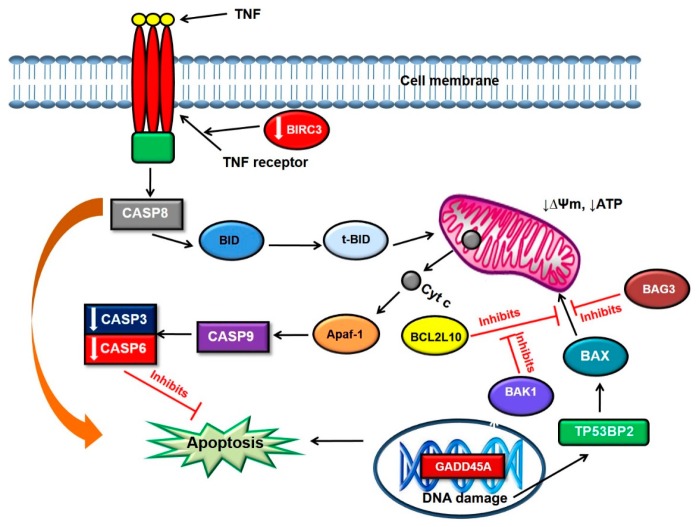
Possible apoptotic pathway induced by photoactivated ZnPcS_mix_ in monolayer cells 24 h post PDT. TNF triggers the extrinsic apoptotic pathway by binding to the TNF receptor and initiating the caspase cascade. CASP8 can directly induce apoptosis or cleave BID into truncated Bid which translocates to the mitochondria. In the mitochondria, BID can result in the release of cytochrome C (cyt c) which activates CASP9 and eventually CASP3. However, in this study, CASP3 and CASP6 were downregulated. BCL2L10 blocks apoptosis induced BAX which in turn is antagonized by BAK 1.

BIRC3 encodes a member of the IAP family of proteins that inhibit apoptosis by binding to TNFR associated factors. Kumari et al. (2007) showed that the PS 5-Aminolevulinic acid (5-ALA) induces apoptosis in human glioblastoma U87MG cells by downregulation of BIRC3 and activation of calpain, caspase-9, and caspase-3 [32]. However, the latter were also found to be downregulated in our study, as well as caspase-6. This is suggestive that the apoptotic cell death observed might follow a caspase independent cell death pathway. The altered expression of genes belonging to the BCL2 family, and TNF signalling in monolayer cells, suggests that there is involvement of both intrinsic and extrinsic apoptotic pathways (Figure 2).

### 3.2. Gene Expression Profiling in the MCTSs

MCTSs with a size of 250 µm revealed upregulation of only 2 genes 24 h post PDT; BCL2/adenovirus E1B 19 kDa interacting protein 3 ‘BNIP3’, and BCL2-related protein A1 ‘BCL2A1’, while there was downregulation of 56 genes (Table 2). BNIP3 is a pro-apoptotic gene capable of homodimerization and heterodimerization with the anti-apoptotic protein BCL2. Activation of BNIP3 leads to localization of BNIP3 in the mitochondria where it causes mitochondrial dysfunction and eventually cell death [33]. Due to their 3D structure, spheroids contain mass-transfer gradients of oxygen and nutrient waste. These metabolic gradients drive proliferation gradients and given sufficient cell numbers and time in culture, spheroids can develop hypoxic cores that can progress to necrosis, closely mimicking what is observed in vivo. BNIP3 expression is highly elevated in hypoxic conditions and PDT is known to induce hypoxia [34]. Growing cancer cells as MCTSs also creates hypoxic conditions in the inner core of the MCTS [35]. A combination of PDT and growing cells as MCTSs might have contributed to the upregulation of the BNIP3 gene in this study. A study conducted by Guo et al. (2001) demonstrated that the over expression of BNIP3 causes apoptotic cell death through a mechanism that does not involve activation of caspase-3 or -9 or cytochrome c release [36]. ROS has also been shown to cause direct activation of BNIP3, and ROS produced after PDT in this study might have contributed to the elevation of BNIP3 [37].

In our previous study, MCTSs with a size of 250 µm showed the highest loss of membrane integrity as revealed by the results of flow cytometry using Annexin V-FITC [21]. It is possible that, in the present study, PDT induced BNIP3 mediated apoptotic cell death in MCTSs with a size of 250 µm, since it was the only gene that was upregulated. BNIP3 may also be associated with mitophagy, but the function of mitophagy during apoptosis has not been resolved. Firstly, mitochondrial outer membrane permeabilization (MOMP) occurs within minutes once activated, whereas mitophagy occurs progressively and secondly, following MOMP, apoptotic caspases inactivate the autophagy induction machinery limiting autophagy induction capacity. Buyen et al. (2011) also demonstrated BNIP3 mediated apoptotic cell death in HT29 cells treated with a chemotherapeutic drug (Cisplatin) [38].

BCL2A1 is an ant-apoptotic gene found to be expressed by proteins in the mitochondrial membrane. Although little is known on how it functions, the pro-survival mechanism can be inhibited [39]. BCL-2, although known to block apoptosis, can promote cell death through an undefined mechanism. BCL-2 has been shown to interact with orphan nuclear receptor ‘Nur77’, which is required for cancer cell apoptosis and can be induced by many anti-neoplastic agents. This interaction results in the conversion of BCL-2 from a protector to a killer, resulting in apoptotic cell death [40].

**Figure 3:**
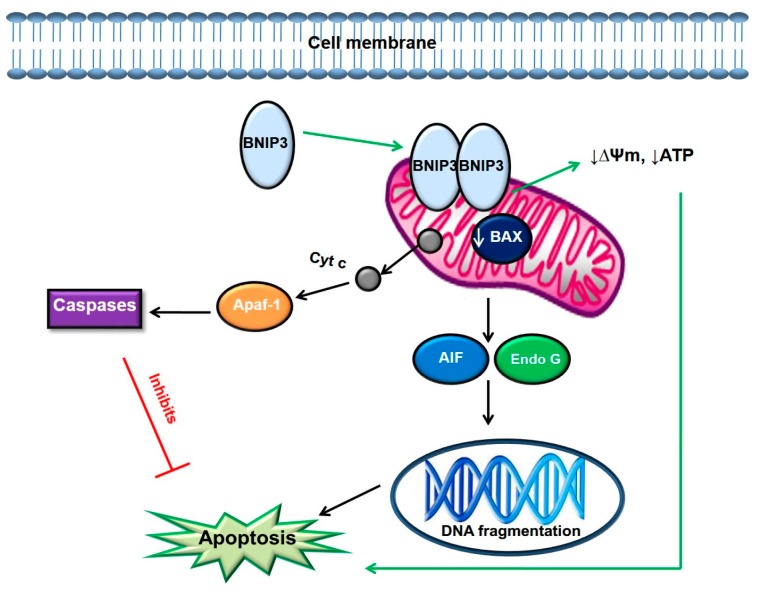
Possible apoptotic pathway induced by photoactivated ZnPcS_mix_ in MCTSs 24 h post PDT. BNIP3 induces apoptosis by dimerization and inserting into the mitochondria. In the mitochondria, BNIP3 can interact with BAX resulting in the permeabilization of the mitochondrial outer membrane which may directly cause apoptosis. Alternatively, permeabilization of the membrane may result in the release of cyt c which activates caspases. However, in this study, gene expression profiling revealed downregulation of caspases, therefore apoptosis was independent of caspases.

In this study, the genes that are known to promote apoptotic cell death were downregulated in MCTSs (250 µm) 24 h post PDT (Table 2). However, upregulation of one pro-apoptotic gene (BNIP3), and the interaction with Nur77, the only anti-apoptotic gene, as described above, induced apoptotic cell death (Figure 3). The downregulation of all anti-apoptotic genes resulted in no genes opposing the pro-apoptotic stimuli induced by BNIP3, thereby rendering cells susceptible to apoptotic cell death.

In MCTSs with a diameter of 500 µm, there was upregulation of only one pro-apoptotic gene (BNIP3) and downregulation of three pro-apoptotic genes—namely BAX, TNFRSF11B, and TNFRSF21. Anti-apoptotic genes that were upregulated were BCL2L10, BNIP3L, CD40LG, and IL10, and there was no downregulation of anti-apoptotic genes. These results suggest that MCTS with a diameter of 500 µm were more resistant to apoptosis. Since BNIP3 was the only pro-apoptotic gene that was upregulated following PDT, the apoptotic cell death seen in MCTSs (500 µm) might be as a result of this gene.

As seen in the smaller MCTSs (250 µm), MCTSs with a diameter of 500 µm also underwent a BNIP3 mediated apoptotic cell death (Figure 2). BNIP3 may also lead to autophagy, however, the mechanism in which it does so is not fully understood [36]. Quinsay et al. (2010) showed that over expression of BNIP3 causes mitochondrial dysfunction and removal of the mitochondria by auto-phagosomes in adult cardiac myocytes [39]. BNIP3 interacts with BCL2, leading to cell death which is not dependent on the BH3 domain thought to be responsible for causing pro-apoptotic activity in other BH3 containing proteins [41].

Interleukin 10 (IL10) is an anti-inflammatory cytokine that prevents the production of cytokines in antigen presenting cells and activated T-cells. It prevents apoptosis by downregulating pro-apoptotic BAX and upregulation of BCL2 [42]. In our study, the downregulation of BAX and upregulation of BCL2 might have been regulated by IL10. A study by Gollnick et al. (1997) showed increased expression of IL10 in skin of mice treated with porfirmer and a 630 nm argon laser, suggesting that IL10 plays a role in cell mediated responses following PDT [43]. Byun et al. (2011) also showed increased expression of IL10 which contributed to the anti-inflammatory effects after PDT in normal human fibroblasts [38].

Nuclear factor kappa B (NF-κB) is implicated in both the suppression and activation of apoptosis. Tumour necrosis factor superfamily member 21 (TNFRSF21) is a pro-apoptotic gene and a member of the TNF-receptor superfamily [44]. Activation of TNFRSF21 in some cell lines leads to apoptosis and activation of the c-Jun N-terminal protein kinase (JNK) and NF-κB pathways which induces apoptosis [45]. The downregulation of TNF superfamily members suggests that PDT did not induce the extrinsic apoptotic pathway in MCTSs.

## 4. Materials and Methods

### 4.1. Monolayer Cell Culture

A human lung cancer cell line (A549, ATCC^®^ CCL-185) was grown in Rosewell Park Memorial Institute 1640 medium (RPMI, Life Technologies, Invitrogen, Johannesburg, South Africa), supplemented with 10% foetal bovine serum (FBS, Gibco, Johannesburg, South Africa), 0.5% penicillin/streptomycin (Gibco) and 0.5% amphotericin-B antifungal (Gibco). Cells were cultured at 37 °C and 5% CO_2_ at 85% humidity.

### 4.2. MCTS Cell Culture

The formation of lung cancer spheroids was initiated by culturing 5 × 10^4^ A549 cells in 75 cm^2^ culture flasks, coated with 1% low melting agarose (Sigma Aldrich, Johannesburg, South Africa). After three days, the resulting aggregates were transferred to 250 mL spinner flasks (Integra Biosciences, Johannesburg, South Africa) containing 150 mL of culture medium. The flasks were placed on a magnetic spinner plate operating at 75 rpm in an incubator set at 37 °C with 5% CO_2_ and 85% humidity. MCTS culture medium was changed three times weekly by allowing the spheroids to settle to the bottom of the spinner flask, carefully removing 100 mL of the medium and replacing with fresh supplemented medium. When spheroids reached approximately 250 µm in diameter, usually after 10 days, and 500 µm diameter after 14 days, they were used for experiments.

### 4.3. Photodynamic Treatment of Monolayer Cells

Once A549 cells were 80% confluent, they were harvested and seeded into 3.4 cm diameter culture dishes for experimental purposes, at a concentration of 2 × 10^5^ cells per culture dish. Cells were allowed to attach to culture dishes for 4 h before incubating with zinc sulfophthalocyanine (ZnPcS_mix_) at a concentration of 10 µM for 24 h. Prior to laser irradiation, cells were washed twice with Hanks Balanced Salt Solution (HBSS, Invitrogen, Johannesburg, South Africa) to remove any unabsorbed ZnPcS_mix_ and fresh media was added. Cells were irradiated using a diode laser emitting at a wavelength of 680 nm and a fluence of 5 J/cm^2^, supplied by the National Laser Centre ‘NLC’ South Africa. All laser irradiations were performed in the dark. The light beam was delivered from the top of the culture dish, with the culture dish lid removed, via fibre optics. Laser irradiation parameters are shown in Table 4. Cells were further incubated, at 37 °C with 5% CO_2_ and 85% humidity, for 24 h before real-time reverse transcription quantitative polymerase chain reaction (RT-PCR) arrays were carried out.

### 4.4. Photodynamic Treatment of MCTS

Fifty A549 spheroids were transferred from spinner flasks to 3.4 cm diameter culture dishes. Spheroids were incubated for 24 h with ZnPcS_mix_ at a concentration of 10 µM. Spheroids were then washed twice in HBSS before laser irradiation and irradiated in the same manner as the monolayer cells above.

### 4.5. Nuclear Morphology

Monolayer cells were seeded onto heat sterilized coverslips in cell culture dishes and treated as in Section 4.3 and incubated 1 or 24 h post PDT. The monolayer cells were stained with Hoechst 33342 (1 μM) by incubation in dye containing growth medium at 37 °C for 5 min. Coverslips were then mounted on a glass slide and fluorescence was visualized as before.

Cryosections of MCTS were prepared by collecting five spheroids using a pipette and placed in 1% eosin stain in order to see the spheroids during sectioning. Spheroids were then washed three times with phosphate buffered saline to remove excess stain. A small amount of shandon cryomatrix was placed on the center of a cryomold and then place inside a cryostat set at −20 °C in order to freeze the shandon cryomatrix. Once the shandon cryomatrix had frozen, spheroids were placed on top of the frozen shandon cryomatrix and then another drop of shandon cryomatrix was placed on top of the spheroids. Once the shandon cryomatrix had frozen, the block was the mounted on a cryostat and sectioned at 10 μm. Cryosections were placed on microscope slides and stained with Hoechst 33342 for 5 min, 1 h or 24 h post PDT.

### 4.6. RNA Isolation and cDNA Synthesis

Total RNA was isolated and purified using the RNeasy Mini Kit (Whitehead Scientific, Qiagen, Johannesburg, South Africa, 74104) with the QIAshredder homogenizers (Whitehead Scientific, Qiagen, 79654). Cells were detached from culture dishes using TrypLE™ Express (Life Technologies, Johannesburg, South Africa, Gibco, Invitrogen, 12605-028) and washed in phosphate buffered saline (PBS) in order to remove all traces of cell culture medium. Cells were lysed with 600 µL RLT buffer, which also inactivates RNases, to ensure purification of the intact RNA. The lysed cells were then loaded onto the QIAcube (Qiagen) with the subsequent release of 30 µL of RNA in under 30 min. Isolated RNA was quantified on the Qubit fluorometer (Invitrogen) using the Quant-iT RNA assay kit (Applied Biosystems, Johannesburg, South Africa, Invitrogen, Q32852). The ratio between the absorbance values at 260 and 280 nm (A_260nm_/A_280nm_) was used to estimate RNA purity using a UV/Vis spectrophotometer. cDNA was synthesized using the QuantiTect Reverse Transcription Kit (Whitehead Scientific, Cape town, South Africa, Qiagen) from 1 µg total RNA. Briefly, samples were treated with gDNA wipe-out buffer and incubated for 2 min at 42 °C. Reverse transcription (RT) master mix was added and samples were incubated for 15 min at 4 °C, and thereafter, 3 min at 95 °C to terminate the reaction. cDNA was stored at minus 20 °C until real time PCR was run.

### 4.7. Gene Expression Profiling

Quantitative Real-Time RT-PCR was performed using the Human Apoptosis RT^2^ Profiler PCR Array (Whitehead Scientific, Johannesburg, South Africa, SABiosciences, PAHS-012Z) which profiles the expression of 84 key genes involves in apoptotic cell death (Table 5). cDNA was thawed on ice and 18 µL of cDNA was added to 93 µL of PCR water, making up the total volume to 111 µL. RT-PCR was performed on the Stratagene MX3000p and a two-step cycling protocol was set on the instrument; 10 min at 95 °C followed by 40 cycles of 15 s at 95 °C and 1 min at 60 °C. A dissociation (melting) curve program was performed using the following parameters; 95 °C for 1 min, 55 °C for 30 s, and 55 °C to 95 °C at a rate of 2 °C per min. Threshold cycle ‘C_t_’ values were exported to an Excel-based Data Analysis Template (available from the SABiosciences website) with the appropriate pathway-focused genes. The C_t_ values of the control and the genes of interest ‘GOI’ were normalized to appropriate endogenous housekeeping ‘HK’ genes. The 2^(−∆∆Ct)^ comparative method was used to determine the fold changes in gene expression between the control (untreated) and PDT treated experimental groups. This was done by comparing the C_t_ values of the samples of interest (PDT treated) with the control i.e., ∆∆C_t_ = ∆C_t_ sample (GOI_treated_ − HKG_treated_) − ∆C_t_ control (GOI_control_ − HKG_control_). Therefore, normalized target gene expression level, or fold change, is 2^(−∆∆Ct)^. Fold change values greater than 1 indicate an upregulated gene, while values less than 1 indicate a downregulated gene (Table 5).

## 5. Statistical Analysis

Experiments were repeated three times (*n* = 3). The Student *t* test was performed by the SABiosciences Excel-based Data Analysis Template, and results were reported as significant if *p* < 0.05.

## 6. Conclusions

The study aimed at identifying which apoptotic genes were involved in cell death of lung cancer cells grown in monolayer versus those grown as tumour spheroids, following photodynamic therapy using a novel photosensitizer (ZnPcS_mix_). This was assessed by measuring the up and downregulation of genes in an apoptotic cell death pathway PCR array. Gene expression analysis showed that photoactivated ZnPcS_mix_ in monolayer cultured cells induced apoptosis through the upregulation of various pro-apoptotic genes, while in MCTSs (with a diameter of 250 and 500 µm), apoptosis was induced through the upregulation of only one gene (BNIP3). In the 250 µm MCTSs, BNIP3 mediated apoptosis was not inhibited by any anti-apoptotic genes. This explains the susceptibility of these MCTSs to PDT compared to monolayer cultures and 500 µm MCTSs where upregulation of pro-apoptotic genes was counteracted by anti-apoptotic genes. Photoactivated ZnPcS_mix_ seems to induce both the intrinsic and extrinsic apoptotic pathway in monolayer cells since there is the involvement of TNF signalling and BCL2 family members. In the MCTSs (250 and 500 µm) the pathway that was followed is inconclusive. However, it was shown that BNIP3—which is located in the cytosol or loosely attached to the mitochondrial outer membrane—played a critical role in the induction of caspase independent apoptosis. The downregulation of the BNIP3 gene has been associated with chemo-resistance in vivo and in our study the BNIP3 gene was upregulated in a tumour model that mimics an in vivo situation. This shows that photoactivated ZnPcS_mix_ may be effective in the treatment of cancer in vivo. Further studies to elucidate the failure of anti-apoptotic genes in MCTSs with a diameter size of 250 µm are required. The understanding of the condition that resulted in the downregulation of anti-apoptotic genes in MCTSs (250 µm) may play a critical role in designing efficient PDT protocols.

## Figures and Tables

**Table 1:** Genes differentially expressed in monolayer A549 cells 24 h post PDT using the Human Apoptosis RT^2^ Profiler PCR Array system. Upregulated genes had a fold change of greater than 1, while downregulated genes had a fold change of less than 1.

Gene Symbol	Description	*p* Value	Fold Change
ABL1	C-abl oncogene 1, non-receptor tyrosine kinase	0.041	1.23
BAG3	BCL2-associated athanogene 3	0.006	1.36
BAK1	BCL2-antagonist/killer 1	0.033	1.41
BCL2L10	BCL2-like 10 (apoptosis facilitator)	0.029	1.68
BID	BH3 interacting domain death agonist	0.035	1.20
BIRC3	Baculoviral IAP repeat containing 3	0.023	−1.94
CASP3	Caspase 3, apoptosis-related cysteine peptidase	0.036	−1.70
CASP5	Caspase 5, apoptosis-related cysteine peptidase	0.028	2.00
CASP6	Caspase 6, apoptosis-related cysteine peptidase	0.045	−1.16
GADD45A	Growth arrest and DNA-damage-inducible, alpha	0.005	1.43
HRK	Harakiri, BCL2 interacting protein (contains only BH3 domain)	0.026	1.96
TNF	Tumour necrosis factor	0.008	3.57
TP53BP2	Tumour protein p53 binding protein, 2	0.019	1.21

**Table 2:** Genes differentially expressed in 250 µm MCTSs 24 h post PDT using the Human Apoptosis RT^2^ Profiler PCR Array system. Upregulated genes had a fold change of greater than 1, while downregulated genes had a fold change of less than 1.

Gene Symbol	Description	*p* Value	Fold Change
AKT1	V-akt murine thymoma viral oncogene homolog 1	0.005	−2.71
APAF1	Apoptotic peptidase activating factor 1	0.001	−1.85
BAD	BCL2-associated agonist of cell death	0.001	−1.55
BAG1	BCL2-associated athanogene	0.034	−1.15
BAX	BCL2-associated X protein	0.000	−1.34
BCL2	B-cell CLL/lymphoma 2	0.041	−1.37
BCL2A1	BCL2-related protein A1	0.022	1.52
BCL2L1	BCL2-like 1	0.004	−1.42
BCL2L11	BCL2-like 11 (apoptosis facilitator)	0.002	−1.88
BFAR	Bifunctional apoptosis regulator	0.001	−1.35
BID	BH3 interacting domain death agonist	0.004	1.10
BIK	BCL2-interacting killer (apoptosis-inducing)	0.038	−1.33
BIRC2	Baculoviral IAP repeat containing 2	0.003	−1.41
BIRC5	Baculoviral IAP repeat containing 5	0.003	−1.45
BIRC6	Baculoviral IAP repeat containing 6	0.005	−1.13
BNIP2	BCL2/adenovirus E1B 19 kDa interacting protein 2	0.010	−1.44
BNIP3	BCL2/adenovirus E1B 19 kDa interacting protein 3	0.009	1.09
BNIP3L	BCL2/adenovirus E1B 19 kDa interacting protein 3-like	0.009	−1.22
BRAF	V-raf murine sarcoma viral oncogene homolog B1	0.001	−1.48
CASP10	Caspase 10, apoptosis-related cysteine peptidase	0.011	−1.88
CASP3	Caspase 3, apoptosis-related cysteine peptidase	0.012	−1.31
CASP4	Caspase 4, apoptosis-related cysteine peptidase	0.000	−1.14
CASP8	Caspase 8, apoptosis-related cysteine peptidase	0.002	−1.55
CASP9	Caspase 9, apoptosis-related cysteine peptidase	0.010	−1.27
CD27	CD27 molecule	0.003	−2.14
CD40	CD40 molecule, TNF receptor superfamily member 5	0.006	−1.31
CD40LG	CD40 ligand	0.022	−1.80
CD70	CD70 molecule	0.001	−1.33
CFLAR	CASP8 and FADD-like apoptosis regulator	0.007	−1.23
CIDEA	Cell death-inducing DFFA-like effector a	0.012	−1.91
CIDEB	Cell death-inducing DFFA-like effector b	0.014	−1.89
CYCS	Cytochrome c, somatic	0.002	−1.92
DAPK1	Death-associated protein kinase 1	0.002	−1.50
DFFA	DNA fragmentation factor, 45 kDa, alpha polypeptide	0.019	−1.30
DIABLO	Diablo, IAP-binding mitochondrial protein	0.028	−1.17
FADD	Fas (TNFRSF6)-associated via death domain	0.032	−1.58
FASLG	Fas ligand (TNF superfamily, member 6)	0.023	−2.03
GADD45A	Growth arrest and DNA-damage-inducible, alpha	0.034	−1.36
IGF1R	Insulin-like growth factor 1 receptor	0.005	−1.87
IL10	Interleukin 10	0.032	−2.02
LTBR	Lymphotoxin beta receptor (TNFR superfamily, member 3)	0.001	−1.15
MCL1	Myeloid cell leukemia sequence 1 (BCL2-related)	0.000	−1.35
NAIP	NLR family, apoptosis inhibitory protein	0.000	−1.75
NFKB1	Nuclear factor of kappa light polypeptide gene enhancer in B-cells 1	0.022	−1.25
NOL3	Nucleolar protein 3 (apoptosis repressor with CARD domain)	0.004	−1.15
TNFRSF10A	Tumour necrosis factor receptor superfamily, member 10a	0.038	−1.92
TNFRSF10B	Tumour necrosis factor receptor superfamily, member 10b	0.025	−1.95
TNFRSF1A	Tumour necrosis factor receptor superfamily, member 1A	0.006	−1.34
TNFRSF25	Tumour necrosis factor receptor superfamily, member 25	0.016	−2.02
TNFSF10	Tumour necrosis factor (ligand) superfamily, member 10	0.000	−2.10
TNFSF8	Tumour necrosis factor (ligand) superfamily, member 8	0.020	−1.83
TP53	Tumour protein p53	0.045	−1.42
TP53BP2	Tumour protein p53 binding protein, 2	0.001	−1.21
TP73	Tumour protein p73	0.030	−2.43
TRADD	TNFRSF1A-associated via death domain	0.001	−1.52
TRAF3	TNF receptor-associated factor 3	0.038	−1.26
XIAP	X-linked inhibitor of apoptosis	0.000	−1.61

**Table 3:** Genes differentially expressed in 500 µm MCTSs 24 h post PDT using the Human Apoptosis RT^2^ Profiler PCR Array system. Upregulated genes had a fold change of greater than 1, while downregulated genes had a fold change of less than 1.

Gene Symbol	Description	*p* Value	Fold Change
BAX	BCL2-associated X protein	0.046	−1.27
BCL2L10	BCL2-like 10 (apoptosis facilitator)	0.034	1.09
BNIP3	BCL2/adenovirus E1B 19 kDa interacting protein 3	0.001	1.71
BNIP3L	BCL2/adenovirus E1B 19 kDa interacting protein 3-like	0.001	1.74
CD40LG	CD40 ligand	0.023	2.76
FASLG	Fas ligand (TNF superfamily, member 6)	0.030	3.52
IL10	Interleukin 10	0.000	3.54
TNFRSF11B	Tumour necrosis factor receptor superfamily, member 11b	0.004	−1.56
TNFRSF21	Tumour necrosis factor receptor superfamily, member 21	0.022	−1.20

**Table 4:** Laser irradiation parameters used in this study.

Parameters	Description/Value
Manufacturer	Oriel Corporation, USA
Wavelength (nm)	680
Wave emission	Continuous wave
Power output (mW)	44.2
Power density (mW/cm^2^)	4.87
Spot size (cm^2^)	9.1
Fluence (J/cm^2^)	5
Duration of exposure	17 min 7 s

**Table 5:** Functional grouping of the SABiosciences Human Apoptosis RT^2^ Profiler PCR Array.

Pathway	Genes
***Induction of apoptosis:***	
Death domain receptors	CRADD, FADD, TNF, TNFRSF10B (DR5)
DNA damage	ABL1, CIDEA, CIDEB, TP53, TP73
Extracellular signals	CFLAR (CASPER), DAPK1, TNFRSF25 (DR3)
Other	BAD, BAK1, BAX, BCL10, BCL2L11, BID, BIK, BNIP3, BNIP3L, CASP1 (ICE), CASP10 (MCH4), CASP14, CASP2, CASP3, CASP4, CASP6, CASP8, CD27 (TNFRSF7), CD70 (TNFSF7), CYCS, DFFA, DIABLO (SMAC), FAS (TNFRSF6), FASLG (TNFSF6), GADD45A, HRK, LTA (TNFB), NOD1 (CARD4), PYCARD (TMS1/ASC), TNFRSF10A, TNFRSF9, TNFSF10 (TRAIL), TNFSF8, TP53BP2, TRADD, TRAF3
***Anti-apoptosis:***	AKT1, BAG1, BAG3, BAX, BCL2, BCL2A1 (Bfl-1/A1), BCL2L1 (BCL-X), BCL2L10, BCL2L2, BFAR, BIRC3 (c-IAP1), BIRC5, BIRC6, BNIP2, BNIP3, BNIP3L, BRAF, CD27 (TNFRSF7), CD40LG (TNFSF5), CFLAR (CASPER), DAPK1, FAS (TNFRSF6), HRK, IGF1R, IL10, MCL1, NAIP (BIRC1), NFKB1, NOL3, RIPK2, TNF, XIAP (BIRC4)
***Regulation of apoptosis:***	
Negative regulation	BAG1, BAG3, BCL10, BCL2, BCL2A1 (Bfl-1/A1), BCL2L1 (BCL-X), BCL2L10, BCL2L2, BFAR, BIRC2 (c-IAP2), BIRC3 (c-IAP1), BIRC6, BNIP2, BNIP3, BNIP3L, BRAF, CASP3, CD27 (TNFRSF7), CD40LG (TNFSF5), CFLAR (CASPER), CIDEA, DAPK1, DFFA, FAS (TNFRSF6), IGF1R, MCL1, NAIP (BIRC1), NOL3, TP53, TP73, XIAP (BIRC4)
Positive regulation	ABL1, AKT1, BAD, BAK1, BAX, BCL2L11, BID, BIK, BNIP3, BNIP3L, CASP1 (ICE), CASP10 (MCH4), CASP14, CASP2, CASP4, CASP6, CASP8, CD40 (TNFRSF5), CD70 (TNFSF7), CIDEB, CRADD, FADD, FASLG (TNFSF6), HRK, LTA (TNFB), LTBR, NOD1 (CARD4), PYCARD (TMS1/ASC), RIPK2, TNF, TNFRSF10A, TNFRSF10B (DR5), TNFRSF25 (DR3), TNFRSF9, TNFSF10 (TRAIL), TNFSF8, TP53, TP53BP2, TRADD, TRAF2, TRAF3
***Death domain proteins:***	CRADD, DAPK1, FADD, TNFRSF10A, TNFRSF10B (DR5), TNFRSF11B, TNFRSF1A, TNFRSF1B, TNFRSF21, TNFRSF25 (DR3), TRADD
***Caspases and regulators:***	
Caspases	CASP1 (ICE), CASP10 (MCH4), CASP14, CASP2, CASP3, CASP4, CASP5, CASP6, CASP7, CASP8, CASP9, CFLAR (CASPER), CRADD, PYCARD (TMS1/ASC)
Caspase activators	AIFM1 (PDCD8), APAF1, BAX, BCL2L10, CASP1 (ICE), CASP9, NOD1 (CARD4), PYCARD (TMS1/ASC), TNFRSF10A, TNFRSF10B (DR5), TP53
Caspase inhibitors	CD27 (TNFRSF7), XIAP (BIRC4)
***Housekeeping genes:***	ACTB, B2M, GAPDH, HPRT1, RPLP0

## Supplementary Materials

Supplementary File 1
