# Supplementary material for: Modes of Cell Death Induced by Photodynamic Therapy Using Zinc Phthalocyanine in Lung Cancer Cells Grown as a Monolayer and Three-Dimensional Multicellular Spheroids
Source: Molecules. 2017 May 16;22(5):791. doi: 10.3390/molecules22050791 (PMC6154333; doi:10.3390/molecules22050791)
Supplement: Supplementary File 1 [file molecules-22-00791-s001.zip › N Hodgkinson - Molecules - Table 5.pdf]

| Gene Symbol | Description | *P* value | Fold change |
|---|---|---|---|
| BAX | BCL2-associated X protein | 0.045885 | -1.27 |
| BCL2L10 | BCL2-like 10 (apoptosis facilitator) | 0.034642 | 1.09 |
| BNIP3 | BCL2/adenovirus E1B 19kDa interacting protein 3 | 0.001461 | 1.71 |
| BNIP3L | BCL2/adenovirus E1B 19kDa interacting protein 3-like | 0.001464 | 1.74 |
| CD40LG | CD40 ligand | 0.023067 | 2.76 |
| FASLG | Fas ligand (TNF superfamily, member 6) | 0.029662 | 3.52 |
| IL10 | Interleukin 10 | 0.000249 | 3.54 |
| TNFRSF11B | Tumour necrosis factor receptor superfamily, member 11b | 0.004189 | -1.56 |
| TNFRSF21 | Tumour necrosis factor receptor superfamily, member 21 | 0.021550 | -1.20 |
