# Supplementary material for: Modes of Cell Death Induced by Photodynamic Therapy Using Zinc Phthalocyanine in Lung Cancer Cells Grown as a Monolayer and Three-Dimensional Multicellular Spheroids
Source: Molecules. 2017 May 16;22(5):791. doi: 10.3390/molecules22050791 (PMC6154333; doi:10.3390/molecules22050791)
Supplement: Supplementary File 1 [file molecules-22-00791-s001.zip › N Hodgkinson - Molecules - Table 4.pdf]

Gene Symbol	Description	*P* value	Fold change
AKT1	V-akt murine thymoma viral oncogene homolog 1	0.004728	-2.71
APAF1	Apoptotic peptidase activating factor 1	0.001058	-1.85
BAD	BCL2-associated agonist of cell death	0.000651	-1.55
BAG1	BCL2-associated athanogene	0.034348	-1.15
BAX	BCL2-associated X protein	0.000025	-1.34
BCL2	B-cell CLL/lymphoma 2	0.040756	-1.37
BCL2A1	BCL2-related protein A1	0.022347	1.52
BCL2L1	BCL2-like 1	0.003947	-1.42
BCL2L11	BCL2-like 11 (apoptosis facilitator)	0.001839	-1.88
BFAR	Bifunctional apoptosis regulator	0.001219	-1.35
BID	BH3 interacting domain death agonist	0.004052	1.10
BIK	BCL2-interacting killer (apoptosis-inducing)	0.037813	-1.33
BIRC2	Baculoviral IAP repeat containing 2	0.003434	-1.41
BIRC5	Baculoviral IAP repeat containing 5	0.002518	-1.45
BIRC6	Baculoviral IAP repeat containing 6	0.005218	-1.13
BNIP2	BCL2/adenovirus E1B 19kDa interacting protein 2	0.010379	-1.44
BNIP3	BCL2/adenovirus E1B 19kDa interacting protein 3	0.008524	1.09
BNIP3L	BCL2/adenovirus E1B 19kDa interacting protein 3-like	0.009845	-1.22
BRAF	V-raf murine sarcoma viral oncogene homolog B1	0.001021	-1.48
CASP10	Caspase 10, apoptosis-related cysteine peptidase	0.011592	-1.88
CASP3	Caspase 3, apoptosis-related cysteine peptidase	0.012060	-1.31
CASP4	Caspase 4, apoptosis-related cysteine peptidase	0.000316	-1.14
CASP8	Caspase 8, apoptosis-related cysteine peptidase	0.002487	-1.55
CASP9	Caspase 9, apoptosis-related cysteine peptidase	0.009549	-1.27
CD27	CD27 molecule	0.003201	-2.14
CD40	CD40 molecule, TNF receptor superfamily member 5	0.005898	-1.31
CD40LG	CD40 ligand	0.022237	-1.80
CD70	CD70 molecule	0.001292	-1.33
CFLAR	CASP8 and FADD-like apoptosis regulator	0.007048	-1.23
CIDEA	Cell death-inducing DFFA-like effector a	0.012164	-1.91
CIDEB	Cell death-inducing DFFA-like effector b	0.013551	-1.89
CYCS	Cytochrome c, somatic	0.002713	-1.92
DAPK1	Death-associated protein kinase 1	0.002531	-1.50
DFFA	DNA fragmentation factor, 45kDa, alpha polypeptide	0.018676	-1.30
DIABLO	Diablo, IAP-binding mitochondrial protein	0.028232	-1.17
FADD	Fas (TNFRSF6)-associated via death domain	0.031986	-1.58
FASLG	Fas ligand (TNF superfamily, member 6)	0.023519	-2.03
GADD45A	Growth arrest and DNA-damage-inducible, alpha	0.033576	-1.36
IGF1R	Insulin-like growth factor 1 receptor	0.005120	-1.87
IL10	Interleukin 10	0.031649	-2.02
LTBR	Lymphotoxin beta receptor (TNFR superfamily, member 3)	0.000921	-1.15
MCL1	Myeloid cell leukemia sequence 1 (BCL2-related)	0.000031	-1.35
NAIP	NLR family, apoptosis inhibitory protein	0.000071	-1.75
NFKB1	Nuclear factor of kappa light polypeptide gene enhancer in B-cells 1	0.022455	-1.25
NOL3	Nucleolar protein 3 (apoptosis repressor with CARD domain)	0.003923	-1.15
TNFRSF10A	Tumour necrosis factor receptor superfamily, member 10a	0.038278	-1.92
TNFRSF10B	Tumour necrosis factor receptor superfamily, member 10b	0.024560	-1.95
TNFRSF1A	Tumour necrosis factor receptor superfamily, member 1A	0.006393	-1.34
TNFRSF25	Tumour necrosis factor receptor superfamily, member 25	0.016203	-2.02
TNFSF10	Tumour necrosis factor (ligand) superfamily, member 10	0.000060	-2.10
TNFSF8	Tumour necrosis factor (ligand) superfamily, member 8	0.020311	-1.83
TP53	Tumour protein p53	0.044978	-1.42
TP53BP2	Tumour protein p53 binding protein, 2	0.001741	-1.21
TP73	Tumour protein p73	0.029935	-2.43

TRADD	TNFRSF1A-associated via death domain	0.001462	-1.52
TRAF3	TNF receptor-associated factor 3	0.038438	-1.26
XIAP	X-linked inhibitor of apoptosis	0.000493	-1.61
