# Supplementary material for: Modes of Cell Death Induced by Photodynamic Therapy Using Zinc Phthalocyanine in Lung Cancer Cells Grown as a Monolayer and Three-Dimensional Multicellular Spheroids
Source: Molecules. 2017 May 16;22(5):791. doi: 10.3390/molecules22050791 (PMC6154333; doi:10.3390/molecules22050791)
Supplement: Supplementary File 1 [file molecules-22-00791-s001.zip › N Hodgkinson - Molecules - Table 3.pdf]

Gene Symbol	Description	*P* value	Fold change
ABL1	C-abl oncogene 1, non-receptor tyrosine kinase	0.041044	1.23
BAG3	BCL2-associated athanogene 3	0.005825	1.36
BAK1	BCL2-antagonist/killer 1	0.032953	1.41
BCL2L10	BCL2-like 10 (apoptosis facilitator)	0.029176	1.68
BID	BH3 interacting domain death agonist	0.035285	1.20
BIRC3	Baculoviral IAP repeat containing 3	0.022851	-1.94
CASP3	Caspase 3, apoptosis-related cysteine peptidase	0.036496	-1.70
CASP5	Caspase 5, apoptosis-related cysteine peptidase	0.027694	2.00
CASP6	Caspase 6, apoptosis-related cysteine peptidase	0.045153	-1.16
GADD45A	Growth arrest and DNA-damage-inducible, alpha	0.004539	1.43
HRK	Harakiri, BCL2 interacting protein (contains only BH3 domain)	0.025595	1.96
TNF	Tumour necrosis factor	0.007676	3.57
TP53BP2	Tumour protein p53 binding protein, 2	0.019300	1.21
