# Supplementary material for: Modes of Cell Death Induced by Photodynamic Therapy Using Zinc Phthalocyanine in Lung Cancer Cells Grown as a Monolayer and Three-Dimensional Multicellular Spheroids
Source: Molecules. 2017 May 16;22(5):791. doi: 10.3390/molecules22050791 (PMC6154333; doi:10.3390/molecules22050791)
Supplement: Supplementary File 1 [file molecules-22-00791-s001.zip › N Hodgkinson - Molecules - Table 2.pdf]

Pathway	Genes
Induction of Apoptosis:	
Death Domain Receptors	CRADD, FADD, TNF, TNFRSF10B (DR5)
DNA Damage	ABL1, CIDEA, CIDEB, TP53, TP73
Extracellular Signals	CFLAR (CASPER), DAPK1, TNFRSF25 (DR3)
Other	BAD, BAK1, BAX, BCL10, BCL2L11, BID, BIK, BNIP3, BNIP3L, CASP1 (ICE), CASP10 (MCH4), CASP14, CASP2, CASP3, CASP4, CASP6, CASP8, CD27 (TNFRSF7), CD70 (TNFSF7), CYCS, DFFA, DIABLO (SMAC), FAS (TNFRSF6), FASLG (TNFSF6), GADD45A, HRK, LTA (TNFB), NOD1 (CARD4), PYCARD (TMS1/ASC), TNFRSF10A, TNFRSF9, TNFSF10 (TRAIL), TNFSF8, TP53BP2, TRADD, TRAF3
Anti-Apoptosis:	
	AKT1, BAG1, BAG3, BAX, BCL2, BCL2A1 (Bfl-1/A1), BCL2L1 (BCL-X), BCL2L10, BCL2L2, BFAR, BIRC3 (c-IAP1), BIRC5, BIRC6, BNIP2, BNIP3, BNIP3L, BRAF, CD27 (TNFRSF7), CD40LG (TNFSF5), CFLAR (CASPER), DAPK1, FAS (TNFRSF6), HRK, IGF1R, IL10, MCL1, NAIP (BIRC1), NFKB1, NOL3, RIPK2, TNF, XIAP (BIRC4).
Regulation of Apoptosis:	
Negative Regulation	BAG1, BAG3, BCL10, BCL2, BCL2A1 (Bfl-1/A1), BCL2L1 (BCL-X), BCL2L10, BCL2L2, BFAR, BIRC2 (c-IAP2), BIRC3 (c-IAP1), BIRC6, BNIP2, BNIP3, BNIP3L, BRAF, CASP3, CD27 (TNFRSF7), CD40LG (TNFSF5), CFLAR (CASPER), CIDEA, DAPK1, DFFA, FAS (TNFRSF6), IGF1R, MCL1, NAIP (BIRC1), NOL3, TP53, TP73, XIAP (BIRC4)
Positive Regulation	ABL1, AKT1, BAD, BAK1, BAX, BCL2L11, BID, BIK, BNIP3, BNIP3L, CASP1 (ICE), CASP10 (MCH4), CASP14, CASP2, CASP4, CASP6, CASP8, CD40 (TNFRSF5), CD70 (TNFSF7), CIDEB, CRADD, FADD, FASLG (TNFSF6), HRK, LTA (TNFB), LTBR, NOD1 (CARD4), PYCARD (TMS1/ASC), RIPK2, TNF, TNFRSF10A, TNFRSF10B (DR5), TNFRSF25 (DR3), TNFRSF9, TNFSF10 (TRAIL), TNFSF8, TP53, TP53BP2, TRADD, TRAF2, TRAF3
Death Domain Proteins:	CRADD, DAPK1, FADD, TNFRSF10A, TNFRSF10B (DR5), TNFRSF11B, TNFRSF1A, TNFRSF1B, TNFRSF21, TNFRSF25 (DR3), TRADD
Caspases and Regulators:	
Caspases	CASP1 (ICE), CASP10 (MCH4), CASP14, CASP2, CASP3, CASP4, CASP5, CASP6, CASP7, CASP8, CASP9, CFLAR (CASPER), CRADD, PYCARD (TMS1/ASC)
Caspase Activators	AIFM1 (PDCD8), APAF1, BAX, BCL2L10, CASP1 (ICE), CASP9, NOD1 (CARD4), PYCARD (TMS1/ASC), TNFRSF10A, TNFRSF10B (DR5), TP53
Caspase inhibitors	CD27 (TNFRSF7), XIAP (BIRC4).

Housekeeping genes:	ACTB, B2M, GAPDH, HPRT1, RPLP0
