# Supplementary material for: Modes of Cell Death Induced by Photodynamic Therapy Using Zinc Phthalocyanine in Lung Cancer Cells Grown as a Monolayer and Three-Dimensional Multicellular Spheroids
Source: Molecules. 2017 May 16;22(5):791. doi: 10.3390/molecules22050791 (PMC6154333; doi:10.3390/molecules22050791)
Supplement: Supplementary File 1 [file molecules-22-00791-s001.zip › N Hodgkinson - Molecules - Table 1.pdf]

**Parameters**	**Description /Value**
Manufacture	Oriel Corporation, USA
Wavelength (nm)	680 nm
Wave emission	Continuous wave
Power output (mW)	44.2 mW
Power density (mW/cm^2)	4.87 mW/cm^2
Spot size (cm^2)	9.1 cm^2
Fluence (J/cm^2)	5 J/cm^2
Duration of exposure	17 min 7 s
