# Supplementary material for: Modes of Cell Death Induced by Photodynamic Therapy Using Zinc Phthalocyanine in Lung Cancer Cells Grown as a Monolayer and Three-Dimensional Multicellular Spheroids
Source: Molecules. 2017 May 16;22(5):791. doi: 10.3390/molecules22050791 (PMC6154333; doi:10.3390/molecules22050791)
Supplement: Supplementary File 1 [file molecules-22-00791-s001.zip › N Hodgkinson - Molecules - Fig 3.pdf]

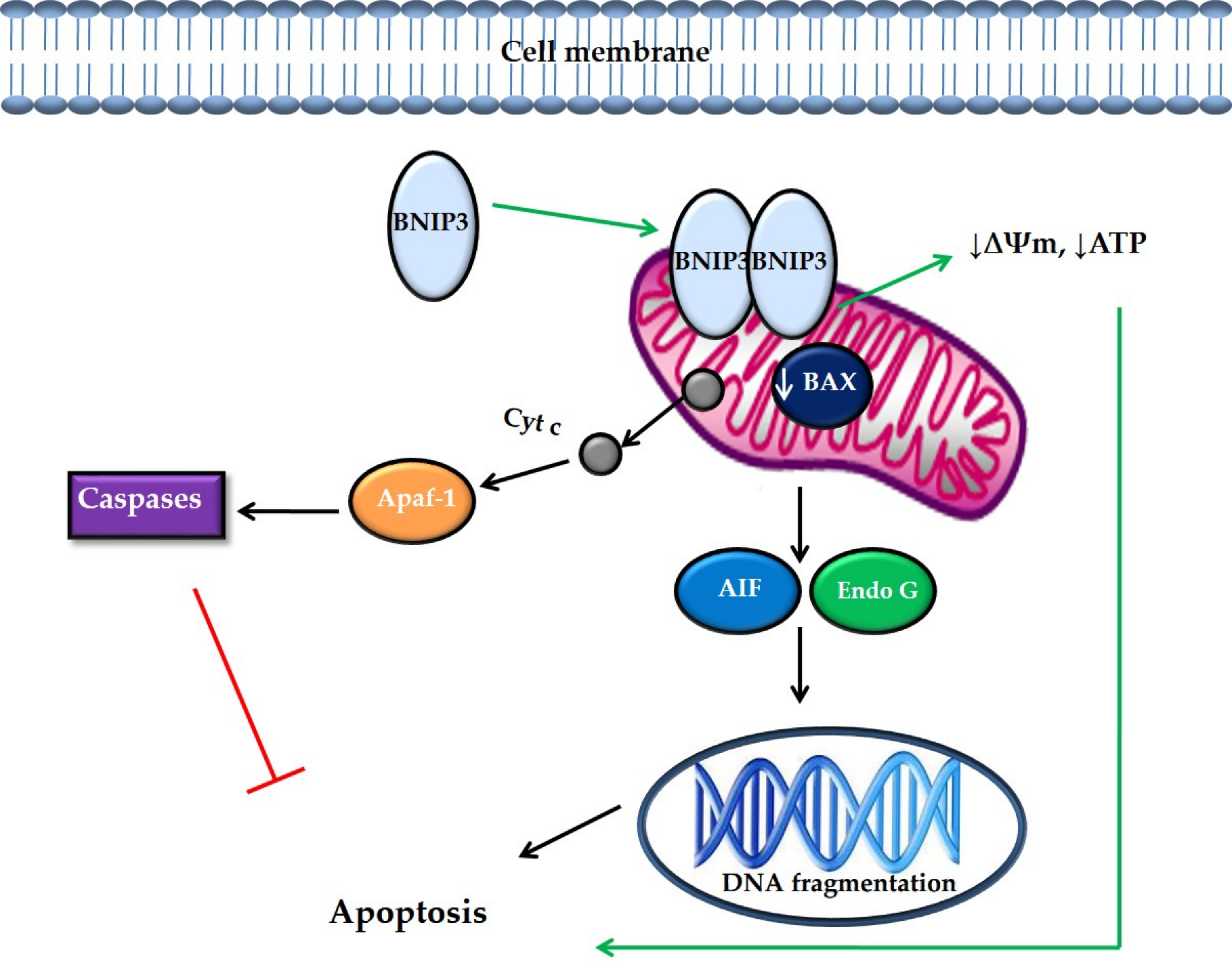
Cell membrane
BNIP3
BNIP3
BNIP3
↓ΔΨm, ↓ATP
↓ BAX
Cyt c
Caspases
Apaf-1
AIF
Endo G
DNA fragmentation
Apoptosis
